# Evidence that SmTetX is not a tetracycline resistance determinant

**DOI:** 10.1128/spectrum.00027-26

**Published:** 2026-04-27

**Authors:** Matthew J. Beech, Maria M. Trush, Edmond C. Toma, Alistair J. M. Farley, Timothy R. Walsh, Christopher J. Schofield

**Affiliations:** 1 Chemistry Research Laboratory, Department of Chemistry and the Ineos Oxford Institute for Antimicrobial Research, University of Oxford 6396, Oxford, United Kingdom; 2 Department of Biology and the Ineos Oxford Institute for Antimicrobial Research, University of Oxford 6396, Oxford, United Kingdom; The Pennsylvania State University, University Park, Pennsylvania, USA

**Keywords:** tetracyclines, antibiotic resistance, drug resistance mechanisms, flavin dependent monooxygenase, *Stenotrophomonas maltophilia*, tigecycline

## Abstract

SmTetX, a flavin-dependent monooxygenase from the opportunistic pathogen *Stenotrophomonas maltophilia*, is structurally related to the tetracycline-degrading Tet(X) resistance enzymes, though displays substantial divergence in active site residues. Here, we provide evidence that recombinant SmTetX is a functional monooxygenase, but the enzyme does not appear to recognize tetracycline antibiotics as substrates and does not catalyze their degradation. Furthermore, SmTetX fails to confer tetracycline resistance when heterologously expressed in *E. coli*. These findings indicate SmTetX is likely not a resistance determinant, presenting an opportunity to identify its physiological substrate and biological role.

**IMPORTANCE:**

The rapid dissemination of *tet(X*) resistance genes threatens the effectiveness of tetracycline antibiotics, including last-resort drugs such as tigecycline. Accurate assignment of functional Tet(X) homologs is therefore important for global antimicrobial resistance surveillance efforts and for selecting targets for the development of therapies that overcome Tet(X) resistance. Here, we demonstrate that SmTetX, a Tet(X)-like enzyme from *Stenotrophomonas maltophilia*, is likely not able to confer resistance to tetracyclines, indicating that its role in tetracycline resistance may have been misattributed. These findings highlight the need for robust experimental validation of novel *tet(X*) genes identified through sequence homology.

## OBSERVATION

**Fig 1:**
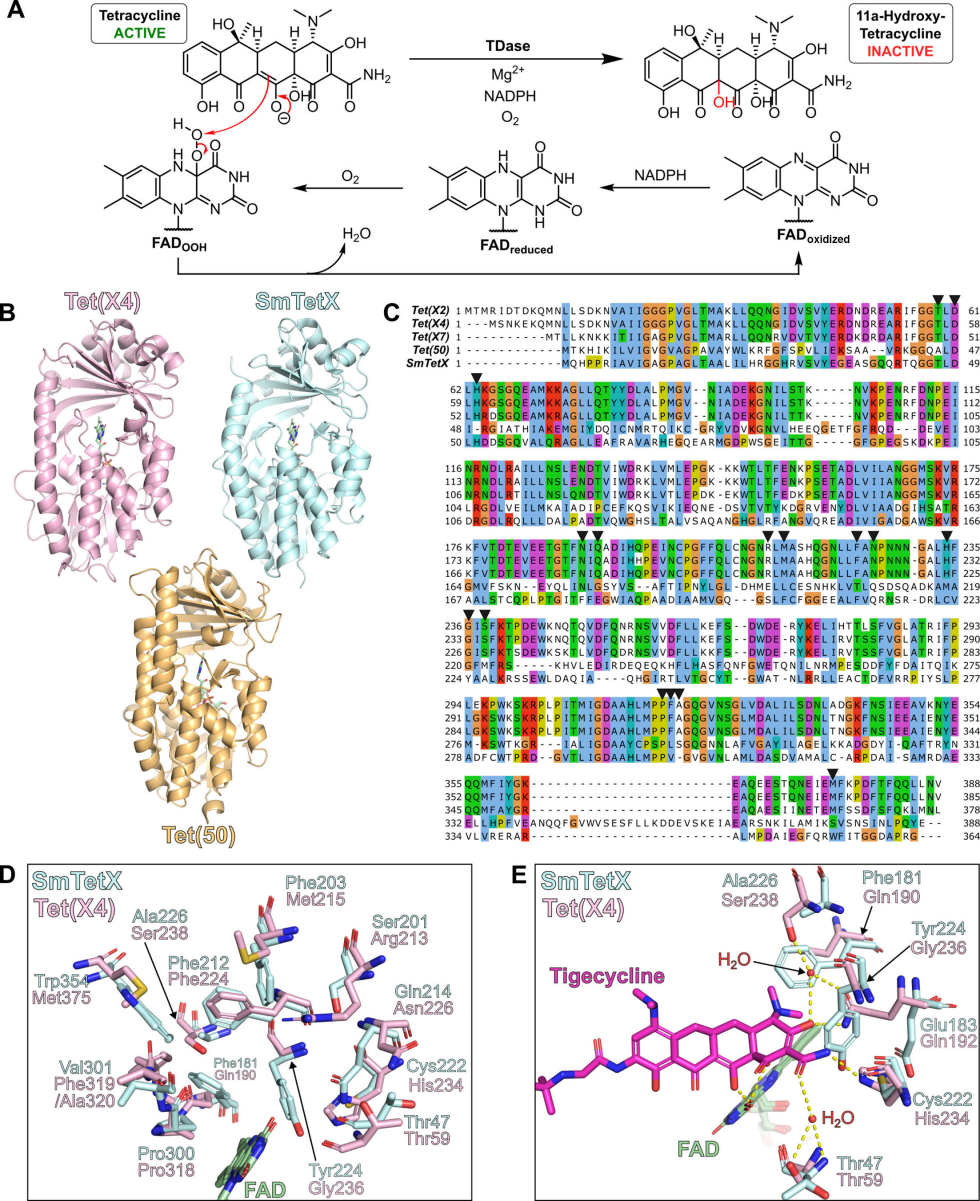
Comparison of SmTetX with tetracycline destructases. (**A**) Outline mechanism of tetracycline inactivation by TDases. (**B**) Comparison of the overall folds of SmTetX (PDB: 8AQ8, blue) with Tet(X4) (PDB: 7EPV, pink) and Tet(50) (PDB: 5TUE, orange). In each case, the flavin cofactor is present (green). (**C**) Amino acid sequence alignment of Tet(X) enzymes, Tet(50), and SmTetX. Tet(X) residues directed toward the substrate binding pocket are highlighted with a black triangle (▼). The alignment was performed using Jalview v2.11.4.1 with the MUSCLE web service and colored using the default ClustalX color scheme. (**D**) View of the active site of Tet(X4) (pink) superimposed with SmTetX (blue). (**E**) View of the active site of tigecycline-Tet(X4) complex (PDB: 7EPW, pink) superimposed with SmTetX, highlighting residues implicated in tigecycline binding. The superimposition was performed in PyMOL v2.5.7 (Schrödinger, LLC).

Tetracycline destructase (TDase) enzymes are an emerging mechanism of tetracycline resistance in Gram-negative pathogens. TDases degrade tetracyclines through C11a-hydroxylation of the tetracycline scaffold via a flavin-dependent monooxygenase (FMO)-type mechanism (Fig. 1A), which is dependent upon enzyme-bound flavin adenine dinucleotide (FAD), reduced β-nicotinamide adenine dinucleotide 2′-phosphate (NADPH), O_2_, and Mg^2+^ ions (1). Genes encoding for type I TDases, also known as the Tet(X) enzymes, are widely and increasingly distributed (2) and often are found on mobile genetic elements, including in co-occurrence with β-lactam and colistin resistance genes (3, 4), leading to the emergence of multidrug-resistant pathogens of clinical concern. Tet(X) enzymes are therefore targets of significant medicinal interest, and information regarding their mechanisms and substrate profiles is valuable in aiding the development of new therapies that overcome tetracycline resistance.

Several Tet(X) variants have been annotated and characterized; they typically possess >85% sequence identity and have only minor differences in their active site residues (5). Recently, a Tet(X) homolog from *Stenotrophomonas maltophilia*, referred to as SmTetX, sharing ~28% sequence identity with Tet(X), was identified through bioinformatics (6). Malý et al. solved a 1.95 Å resolution crystal structure of *apo*-SmTetX (PDB: 8AQ8), revealing close structural homology in the overall fold of SmTetX compared to that of other characterized type I TDases (Fig. 1B), such as Tet(X2), Tet(X4), and Tet(X7) (*C_α_* RMSD = 1.54 Å, 1.35 Å, and 1.61 Å, respectively). SmTetX also lacks the additional C-terminal α-helix characteristic of type II TDases such as Tet(50) (7). Despite similarities in their overall folds, SmTetX manifests substantial differences in its active site residues compared to Tet(X) homologs (Fig. 1C and D), suggesting a different tetracycline binding mode may be necessary to accommodate the substrate in the SmTetX active site. In particular, replacement of Tet(X4) residues Ser238, Gln192, and His234 by Ala226, Glu183, and Cys222 in SmTetX, respectively, would appear likely to disrupt key hydrogen bonds formed with the A-ring of tigecycline in its crystallographically observed binding mode (8) (Fig. 1E). Additionally, replacement of Gln190 and Gly236 of Tet(X4) with Phe181 and Tyr224 in SmTetX introduces sterically bulky aromatic residues into the active site which would appear to create clashes with the tetracycline A-ring dimethylamino and carboxamide groups.

*Stenotrophomonas maltophilia* is a rapidly evolving, often multidrug-resistant, Gram-negative opportunistic pathogen that can cause various nosocomial infections, particularly in immunocompromised, cystic fibrosis, cancer, and HIV/AIDS patients (9). Resistance to tetracyclines in *S. maltophilia* has been primarily attributed to the activity of multiple efflux pumps, including SmrA, SmeDEF, SmeVWX, and SmeGH (9, 10). We were, therefore, interested to investigate the substrate scope of SmTetX and how its apparently unusual active site residues might affect tetracycline recognition and impact inhibitor design for future drug discovery.

**Fig 2:**
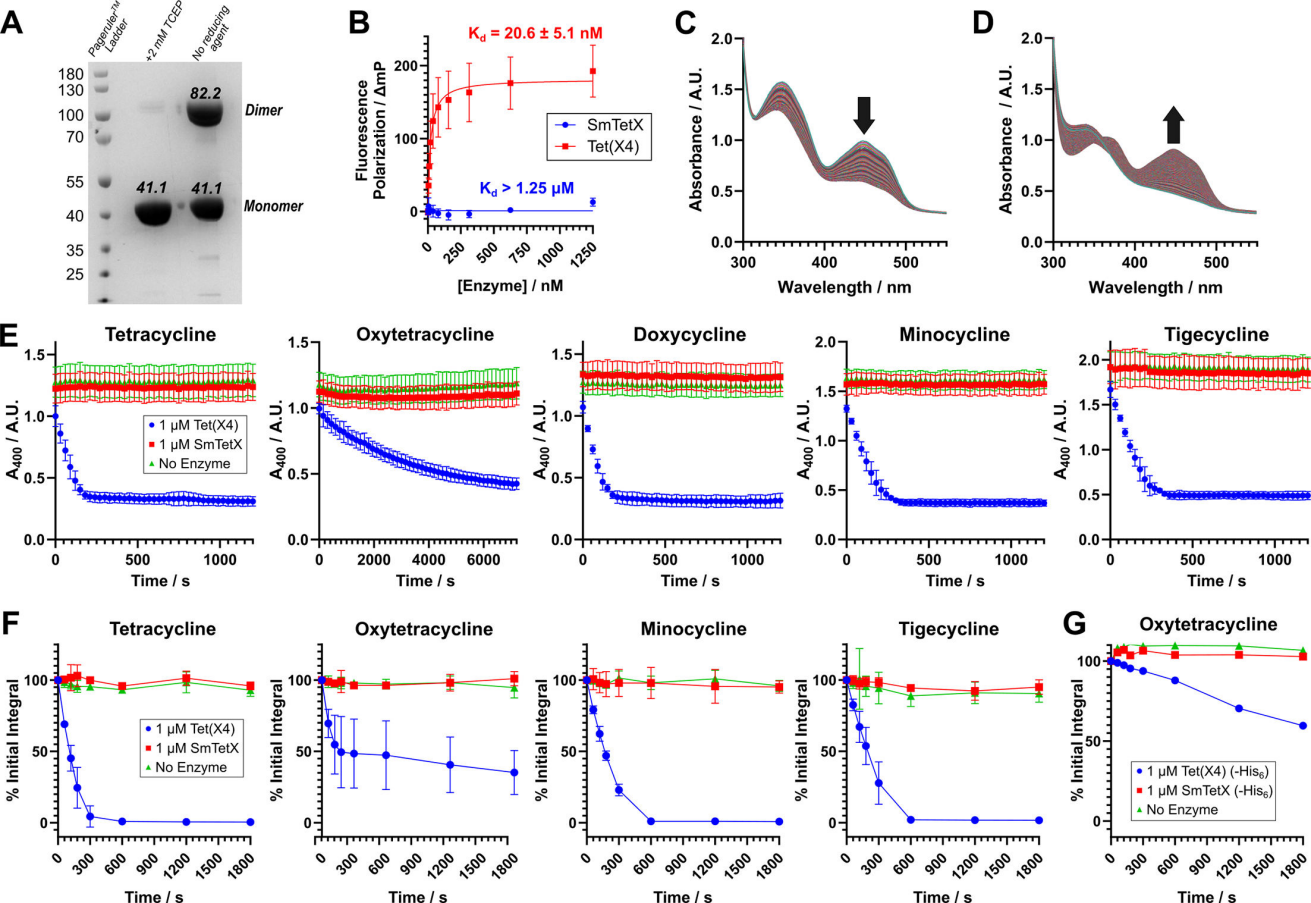
Investigating the activity of recombinant SmTetX. (**A**) SDS-PAGE gel demonstrating identity and purity of recombinant SmTetX (Lane 1: PageRuler Reference Ladder; Lane 2: SmTetX treated with 2 mM TCEP; Lane 3: SmTetX following Ni-affinity chromatography). (**B**) Fluorescence polarization binding assays of SmTetX and Tet(X4) with a TAMRA-glycine-minocycline fluorescent probe (*n* = 4). (**C**) Absorbance spectra demonstrating the reduction of the SmTetX cofactor FAD when exposed to NADPH under anaerobic conditions, confirming FMO activity. (**D**) Absorbance spectra demonstrating the re-oxidation of the reduced flavin cofactor under aerobic conditions, evidencing the potential of SmTetX to form the reactive C4a-hydroperoxyflavin species. (**E**) Absorbance measurements at 400 nm demonstrating degradation of tetracycline analogs by Tet(X4) and SmTetX compared to a no enzyme negative control (*n* = 4). (**F**) Normalized UPLC peak integrals showing degradation of tetracycline analogs by Tet(X4) and SmTetX compared to a no enzyme negative control (*n* = 3). (**G**) Normalized UPLC peak integrals demonstrating degradation of oxytetracycline by SmTetX and Tet(X4) with the His_6_-tag cleaved, compared to Tet(X4) and a no enzyme control. Error bars in all cases represent the standard deviation from independent repeats.

To investigate its function, recombinant SmTetX was produced in *E. coli* and purified according to the previously reported conditions (6), initially without removal of the N-terminal His_6_-tag (Fig. 2A). We observed covalent homodimer formation during purification, which is also observed in the reported crystal structure of SmTetX (6), likely an artefact as a result of heterologous expression and aerobic purification. Dimerization could be reversed by overnight treatment with the disulfide reducing agent tris(2-carboxyethyl)phosphine (TCEP) to give monomeric SmTetX, which was used in enzyme assays. We initially attempted to characterize SmTetX binding to tetracyclines by employing our reported fluorescence polarization binding assay (11). However, we observed no clear change in the polarization response in the presence of up to 1.25 µM of SmTetX (Fig. 2B), a concentration up to 100-fold higher than the K_d_ values measured for Tet(X) enzymes (11), indicating minimal affinity for the enzyme.

To test whether the produced SmTetX enzyme was folded correctly and was an active FMO, its reductive and oxidative half-reactions were investigated. The reductive half-reaction was probed by reaction of SmTetX with NADPH under anaerobic conditions (Fig. 2C). Time-dependent disappearance of the FAD absorbance peak at 452 nm was observed, confirming that the flavin cofactor of SmTetX can be reduced by NADPH. Reintroduction of oxygen by removal of the airtight seal and incubation in ambient aerobic conditions caused the appearance of a new absorbance peak at 380 nm, consistent with formation of the C4a-hydroperoxyflavin intermediate, with subsequent reappearance of the oxidized FAD peak at 452 nm (Fig. 2D). These observations indicate that under the reaction conditions used SmTetX should be able to act as a flavin-dependent monooxygenase, given the appropriate substrate. We then attempted to monitor the turnover of several tetracyclines by the SmTetX enzyme utilizing reported activity assays using an absorbance readout (11, 12). No significant decay of the A_400_ signal was observed with the putative substrates tetracycline, oxytetracycline, doxycycline, minocycline, or tigecycline in the presence of 1 µM SmTetX compared to the no enzyme controls with an excess of NADPH, indicating no turnover (Fig. 2E). By contrast, efficient reaction was observed with the type I TDase Tet(X4) under the same conditions (Fig. 2E).

We then investigated potential turnover of tetracyclines by SmTetX using a UPLC-based assay using reported conditions (11), with a modified quenching and analysis procedure where enzymes were incubated with tetracyclines, NADPH, and MgCl_2_ then quenched at intervals by addition of 1% (vol/vol) aqueous formic acid solution containing 500 µM Fmoc-glycine. The time-dependent degradation of the starting tetracycline peak was measured by normalizing the peak integral against the Fmoc-glycine internal reference standard. Consistent with the absorbance assay results, turnover was not observed with SmTetX, but was observed in concurrent measurements with Tet(X4) (Fig. 2F). For completeness, we also tested the activity of the enzyme with the His_6_-tag-cleaved SmTetX and Tet(X4) proteins (Fig. 2G). Tag-cleaved Tet(X4) was still able to hydroxylate oxytetracycline, indicating that the N-terminal tag was not impeding activity, while the tag-cleaved SmTetX remained inactive toward oxytetracycline.

Finally, we attempted to measure the susceptibility of bacteria expressing SmTetX to tetracycline antibiotics by heterologous overexpression in *E. coli*, based on methods developed for susceptibility testing with Tet(X4) (11). The *smtetX* gene was subcloned into the pBAD-TOPO vector by topoisomerase-based cloning, enabling expression to be controlled by induction with ʟ-arabinose in *E. coli* TOP10 cells. MICs for tetracyclines were measured using broth microdilution methodology. Overexpression of SmTetX did not enhance resistance to any of the tested tetracyclines compared to controls with no ʟ-arabinose, *E. coli* TOP10 with no vector and *E. coli* TOP10 with an empty pBAD-TOPO vector, suggesting that SmTetX may not contribute to tetracycline resistance (Table 1). In contrast, overexpression of Tet(X4) under the same experimental conditions resulted in increased MICs for tetracycline, oxytetracycline, doxycycline and tigecycline of between 8- and 32-fold compared to the no ʟ-arabinose control or between 32- and 128-fold compared to the empty pBAD plasmid control, consistent with previously reported values for similar model strains expressing Tet(X) variants (13, 14).

**TABLE 1:** Minimum inhibitory concentration (MIC) for selected tetracyclines against *E. coli* TOP10 containing plasmids pBAD-TOPO, pBAD-TOPO-SmTetX, or pBAD-TOPO-Tet(X4) induced with ʟ-arabinose^*a*^

Strain	ʟ-arabinose/% (wt/vol)	MIC/µg mL^−1^			
		TET^*b*^	OTC^*c*^	DOX^*d*^	TIG^*e*^
*Escherichia coli* ATCC 25922	0	1	1	1	≤0.06
*Escherichia coli* TOP10	0	1	1	0.5–1	≤0.06
*Escherichia coli* TOP10 pBAD-TOPO	0.2	2	1	0.5	0.13
	0.02	2	1	0.5	0.13
	0.002	2	1	0.5	0.13
	0.0002	2	1	0.5	0.13
	0	2	1	0.5	0.13
*Escherichia coli* TOP10 pBAD-TOPO-SmTetX	0.2	1	1	0.5–1	≤0.06
	0.02	1	1	0.5–1	≤0.06
	0.002	1	1	0.5–1	≤0.06
	0.0002	1	1	0.5–1	≤0.06
	0	1	1	0.5–1	≤0.06
*Escherichia coli* TOP10 pBAD-TOPO-Tet(X4)	0.2	>64	>64	16	8-16
	0.02	64	>64	16	8
	0.002	8	8–16	2	2
	0.0002	8	8	2	1
	0	4–8	8	2	0.5

^*a*^ MIC data represent results from three independent experiments.

^*b*^ TET, tetracycline.

^*c*^ OTC, oxytetracycline.

^*d*^ DOX, doxycycline.

^*e*^ TIG, tigecycline.

The biochemical and microbiological data presented here imply that although SmTetX is likely a flavin-dependent monooxygenase as proposed (6), it is likely not a tetracycline resistance determinant. It appears unlikely that SmTetX accepts tetracycline antibiotics as substrates, and its role as a flavin-dependent monooxygenase in *S. maltophilia* remains unknown. These observations present a new research opportunity to identify the natural substrate of SmTetX and its biological relevance in *S. maltophilia*. It may be possible to investigate the substrate recognition of SmTetX through detailed bioinformatics or, as a high-resolution structure has been reported (6), through *in silico* predictions.

## Data Availability

The authors declare that all relevant data are available in the manuscript and supplemental material. Requests for materials should be made to Christopher J. Schofield (christopher.schofield@chem.ox.ac.uk).

## SUPPLEMENTAL MATERIAL

Supplemental material spectrum.00027-26-s0001.docx Fig. S1 and S2.

ASM does not own the copyrights to Supplemental Material that may be linked to, or accessed through, an article. The authors have granted ASM a non-exclusive, world-wide license to publish the Supplemental Material files. Please contact the corresponding author directly for reuse.
