# Supplementary material for: Evidence that SmTetX is not a tetracycline resistance determinant
Source: Microbiol Spectr. 2026 Apr 27;14(6):e00027-26. doi: 10.1128/spectrum.00027-26 (PMC13228020; doi:10.1128/spectrum.00027-26)
Supplement: Supplemental material — Fig. S1 and S2. [file spectrum.00027-26-s0001.docx]

**Supplementary Information**

**Evidence that SmTetX is not a tetracycline resistance determinant**

Matthew J. Beech^a‡^*, Maria M. Trush^b‡^, Edmond C. Toma^a^, Alistair J. M. Farley, Timothy R. Walsh^b^, and Christopher J. Schofield^a^*

^a^Chemistry Research Laboratory, Department of Chemistry and the Ineos Oxford Institute for Antimicrobial Research, University of Oxford, Oxford, OX1 3TA, UK.

^b^Department of Biology and the Ineos Oxford Institute for Antimicrobial Research, University of Oxford, Oxford, OX1 3RE, UK

^‡^These authors contributed equally to this work.

*Corresponding authors: Christopher J. Schofield ([christopher.schofield@chem.ox.ac.uk](mailto:christopher.schofield@chem.ox.ac.uk)) & Matthew J. Beech ([matthew.beech@chem.ox.ac.uk](mailto:matthew.beech@chem.ox.ac.uk))

**Experimental Details**

**Protein overproduction and purification**

Highly purified Tet(X4) was produced as reported (11). SmTetX was overproduced and purified in a similar manner to that reported (6). Thus, Lemo21(DE3) *E. coli* cells (New England Biolabs) were transformed with the SmTetX-pET-28b(+) plasmid. Cells were grown at 37 °C in 2x YT media supplemented with 50 µg/mL kanamycin to an A_600_ of 0.6. Recombinant protein production was induced by addition of 0.5 mM isopropyl β-D-1-thiogalactopyranoside. Cells were incubated overnight at 16 °C and harvested by centrifugation (8000 rpm, 10 mins). Cells were resuspended in buffer A (50 mM Tris (pH 8.0), 500 mM NaCl and 20 mM imidazole) supplemented with a cOmplete™ Protease Inhibitor tablet (Roche), 1 mg L^-1^ lysozyme (Sigma Aldrich) and 1 mg L^-1^ DNAase I (Roche), then lysed using a cell disruptor (Constant Systems, 20 kpsi). The cell lysates were clarified by centrifugation and the supernatant was loaded onto a 5 mL HisTrap column (Cytiva Life Sciences). The column was washed with buffer A (15 column volumes (CV)), then eluted with a linear gradient of buffer B (50 mM Tris (pH 8.0), 500 mM NaCl and 300 mM imidazole) in buffer A over 20 CV. Fractions containing the expected protein (as determined by SDS-PAGE gel analysis) were pooled, concentrated and loaded onto a size-exclusion chromatography column (Superdex-75 16/60, GE Healthcare) using 50 mM Tris (pH 8.0), 150 mM NaCl as the buffer. Dimerisation was observed by SDS-PAGE analysis following this step, so tris(2-carboxyethyl)phosphine (TCEP) was added to a final concentration of 2 mM with overnight incubation at 4 °C. The monomeric protein was buffer exchanged into buffer containing 50 mM Tris (pH 8.0), 150 mM NaCl, 5 mM DTT by several rounds of concentration and dilution in a 30 kDa MWCO centrifugal filter (Merck Millipore). When the TCEP concentration was calculated to be approximately < 0.1 µM, the protein was aliquoted, flash-cooled in liquid N_2_, then stored at -80 °C.

To obtain the thrombin-cleaved protein, an aliquot of SmTetX or Tet(X4) was thawed (~1 mg), diluted to 50 μM with thrombin cleavage buffer (Merck) and 2 units of Restriction Grade thrombin (Merck) was added. The protein was incubated at 4 °C for 16 h. After cleavage was confirmed by SDS-PAGE analysis (Supplementary Figure 1) and protein-observed mass spectrometry (Supplementary Figure 2), the mixture was added to a His SpinTrap™ (Merck) column and the cleaved material eluted according to the manufacturer’s protocol. The protein solution was buffer exchanged into buffer containing 50 mM Tris (pH 8.0), 150 mM NaCl, and 5 mM DTT by rounds of concentration and dilution in a 30 kDa MWCO centrifugal filter before use.

**Fluorescence polarisation binding assays**

Fluorescence polarisation measurements were conducted as reported (11). In brief, 2-fold serial dilution of enzymes (4-1250 nM final concentrations, with a no enzyme negative control) in buffer containing 100 mM Tris (pH 7.0) with 0.01% Triton X-100 were added to a non-binding, 384-well black plate (Greiner Bio-One) in quadruplicate. A mixture containing the TRITC-Glycine-Minocycline fluorescent probe (11), FAD and MgCl_2_ (final concentrations of 25 nM, 1 µM and 5 mM respectively) was then dispensed using an Integra Biosciences WellJET dispenser equipped with an 8-channel cassette and incubated at ambient temperature for 30 min. Fluorescence polarisation was then measured using a PHERAstar FS microplate reader (BMG Labtech) equipped with an FP optic module (λ_Ex_ = 540 ± 20 nm, λ_Ex_ = 590 ± 20 nm, 200 flashes per well). ΔmP was calculated and plotted as a function of enzyme concentration and fit using a one-site specific binding model (GraphPad Prism 10.1.2) to calculate the dissociation constant, K_d_.

**Monitoring reductive and oxidative half-reactions**

N_2_ was bubbled through a sealed vial of 100 mM TAPS buffer (pH 8.5) for 30 mins to remove O_2_. An aliquot of SmTetX was allowed to equilibrate in an anaerobic chamber (Belle Technologies, O_2_ < 10 ppm) for 30 mins prior to use. MgCl_2_ and NADPH were dissolved in deoxygenated buffer in the anaerobic chamber. SmTetX, MgCl_2_ and buffer were mixed in a quartz cuvette (90 µL) and fitted with a Suba-Seal® (Sigma Aldrich) in the anaerobic chamber. The NADPH/MgCl_2_ solution was transferred to a separate vial, which was fitted with a Suba-Seal®. The UV spectrum of the oxidised FAD was recorded. The NADPH solution (10 µL) was added via a gas-tight syringe (Hamilton) and UV spectra were recorded over time (Agilent Cary 3500 UV-vis Compact Peltier spectrometer, 300-550 nm, ~1.5 s intervals, 5 min total) to monitor the reductive half reaction. To monitor the oxidative reaction, the Suba-Seal® was removed from the cuvette to introduce oxygen and UV spectra were recorded over time (Agilent Cary 3500 UV-vis Compact Peltier spectrometer, 300-550 nm, ~1 s intervals, 5 min total). The final concentrations in the reaction mixture: were 100 µM SmTetX, 10 mM MgCl_2_ and 100 µM NADPH.

**Absorbance-based tetracycline degradation assay (11)**

FAD, MgCl_2_ and purified enzyme (SmTetX, Tet(X4) or buffer for a no enzyme control) in 100 mM TAPS (pH 8.5) buffer, 50 µL, were dispensed into a 96-well half-area clear plate (Greiner Bio-One) was dispensed (Integra Biosciences WellJET). Tetracycline analogues dissolved in DMSO were added using a multichannel pipette and the solution was mixed. NADPH in buffer (50 µL) was then dispensed. Final concentrations were: 1 µM enzyme, 1 µM FAD, 5 mM MgCl_2_, 500 µM NADPH, 100 µM tetracycline analogue, 2% DMSO. Absorbance at 400 nm (common tetracycline chromophore) was measured using a CLARIOstar microplate reader (BMG Labtech) over the course of 2 h at ambient temperature.

**UPLC-based tetracycline degradation assay (11)**

FAD, MgCl_2_ and purified enzyme (SmTetX, Tet(X4) or buffer for a no enzyme control) in 100 mM TAPS (pH 8.5) buffer, 50 µL, were added to a 96-well 2 mL Masterblock® plate (Greiner Bio-One). Tetracycline analogues dissolved in DMSO were added and mixed. A solution of NADPH in TAPS buffer was added using a multichannel pipette. Final concentrations were: 1 µM enzyme, 1 µM FAD, 5 mM MgCl_2_, 500 µM NADPH, 100 µM tetracycline analogue. 2%_v/v_ DMSO. At time intervals, 100 µL of reaction mixture was quenched into 10 µL of a 10%_v/v_ aqueous solution of formic acid containing 500 µM Fmoc-glycine in a 96-well skirted PCR plate (Sarstedt). Samples were analysed using an ACQUITY H-Class PLUS UPLC instrument (Waters) equipped with a pre-equilibrated ACQUITY BEH C18 column (20 × 50 mm, 1.7 μM pore size, Waters) using a gradient of 1–50%_v/v_ acetonitrile (with 0.1%_v/v_ formic acid) in water (with 0.1%_v/v_ formic acid) over 1.83 minutes at a flow rate of 0.5 mL min^−1^. Absorbance was measured using a TUV detector at a wavelength of 254 nm. Instrument control and data processing were performed using MassLynx V4.1 software (Waters). Data was normalised relative to the integral of the Fmoc-Glycine internal standard and calculated as a percentage of the normalised integral at t = 0 s.

**Plasmid construction**

The *smtetX* gene (WP_049406473) was synthesised by Gene Universal with optimisation for *E. coli* codons and inserted using *NdeI* and *BamHI* sites into a pET-28b(+) vector, to produce recombinant protein with an *N*-terminal His_6_-tag and thrombin cleavage site. For l-arabinose-inducible expression, the gene was subcloned from pET-28b(+) into the pBAD-TOPO vector via TOPO cloning. The desired gene was amplified using primers 5′-CATATGCAGCACCCGCCGCGTATC-3′ and 5′-GGATCCTCAACCACGCGGCG-3′ using the onetaq polymerase (New England Biolabs), purified from a 1% agarose gel using a GeneJET gel extraction kit (Thermo-Fisher) and the A-tailed product ligated into pBAD-TOPO using a kit (Invitrogen) according to the manufacturer’s protocol. The empty pBAD-TOPO control plasmid was generated from the pBAD-TOPO-SmTetX plasmid via in-frame deletion mutagenesis with the primers 5′-GGATCCAAGGGCGAG-3′ and 5′- GGGTATGTATATCTCCTTCTTAAAG-3′ using a Q5 site-directed mutagenesis kit (NEBiolabs). Blunt-end ligation was performed according to the manufacturer’s protocol. The plasmid pBAD-TOPO-Tet(X4) was used as described previously (11).

**Minimum Inhibitory Concentration (MIC) Determination**

Plasmids were transformed into TOP10 *E. coli* cells (Invitrogen) and transformants were selected on LB agar plates containing ampicillin (100 mg L^-1^) and 0.02%_w/v_ l-arabinose after incubation at 37 °C for 24 h. MIC testing was performed in round-bottom 96-well microtiter plates using a modified broth microdilution protocol following CLSI guidelines (3). Susceptibility testing was carried out in freshly prepared cation-adjusted Mueller-Hinton Broth (CAMHB) by the two-fold serial dilution method with an inoculum of ~ 0.75 x 10^6^ cells mL^-1^ (15). Oxytetracycline, doxycycline, tetracycline and tigecycline were obtained from Sigma-Aldrich, UK. All antibiotics were dissolved in DMSO. *Escherichia coli*ATCC 25922 and plasmid-free *Escherichia coli* TOP10 were used as controls. Induction of SmTetX and Tet(X4) was maintained at different l-arabinose concentrations to create 0.2%_w/v_, 0.02%_w/v_, 0.002%_w/v_, and 0.0002%_w/v_ dilutions. MICs were reported as the lowest antibiotic concentrations that inhibited visible bacterial growth after an overnight incubation at 37 °C. MIC data represent results from three independent experiments.

**Protein-Observed Mass Spectrometry**

Proteins diluted to 5 µM were analysed using a Waters XeVo G2-S Q-TOF mass spectrometer (Waters) coupled to an ACQUITY UPLC (Waters) fitted with a ProSwift RP-4H (1 x 50 mm, ThermoFisher Scientific). Protein was eluted using a gradient of 5-95%_v/v_ acetonitrile in water (both with 0.1% formic acid) over 8 minutes. Deconvolution was performed in MassLynx v4.1.

**Supplementary Figures**

**
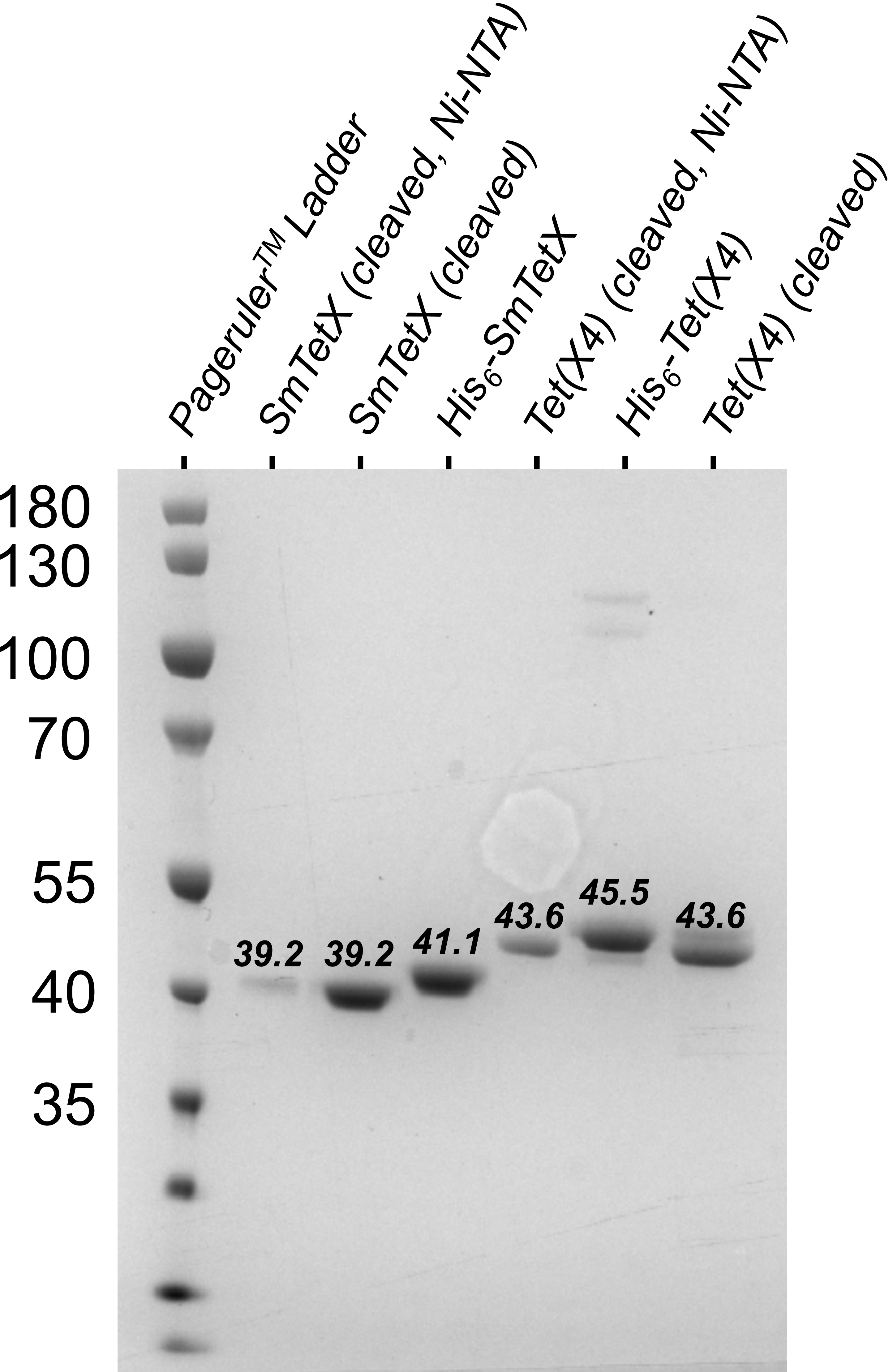
**

**Supplementary Figure 1 |** 4-12% SDS-PAGE gel (Invitrogen) demonstrating the successful thrombin cleavage of purified recombinant SmTetX and Tet(X4) prior to biochemical assays (Lane 1: PageRuler Reference Ladder; Lane 2: SmTetX following thrombin cleavage and reverse Ni-affinity chromatography; Lane 3: SmTetX following thrombin cleavage; Lane 4: His_6_-SmTetX; Lane 5: Tet(X4) following thrombin cleavage and reverse Ni-affinity chromatography; Lane 6: His_6_-Tet(X4); Lane 7: Tet(X4) following thrombin cleavage).


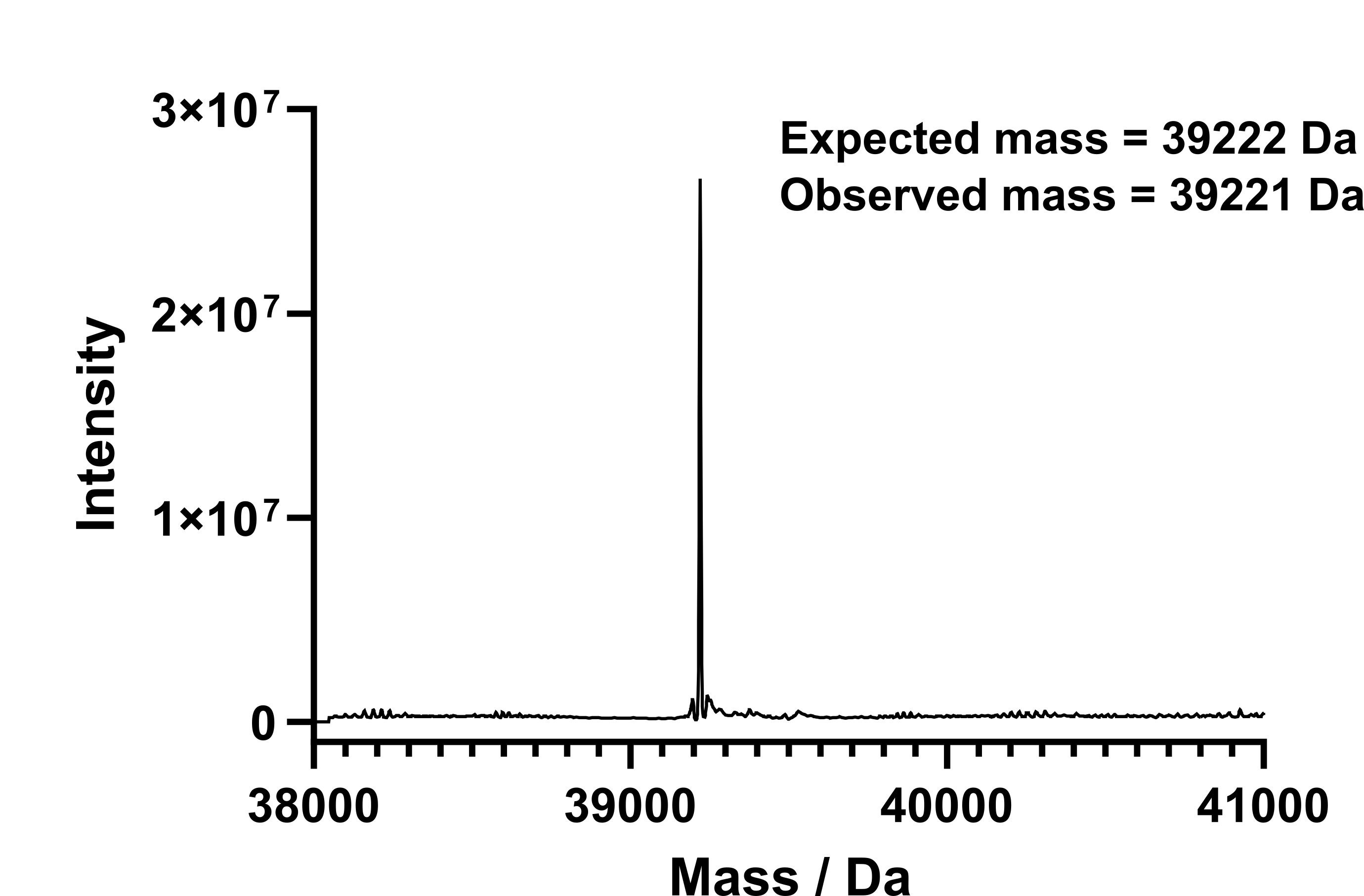


**Supplementary Figure 2 |** Deconvoluted mass spectrum demonstrating the identity of thrombin-cleaved SmTetX.

**References**

6. Malý M, Kolenko P, Stránský J, Švecová L, Dušková J, Koval’ T, Skálová T, Trundová M, Adámková K, Černý J, Božíková P, Dohnálek J. 2023. Tetracycline-modifying enzyme *Sm* TetX from *Stenotrophomonas maltophilia*. Acta Crystallogr F Struct Biol Commun 79:180–192.

11. Beech MJ, Toma EC, Smith HG, Trush MM, Ang JHJ, Wong MY, Wong CHJ, Ali HS, Butt Z, Goel V, Duarte F, Farley AJM, Walsh TR, Schofield CJ. 2025. Binding assays enable discovery of Tet(X) inhibitors that combat tetracycline destructase resistance. Chem Sci 16:9691–9704.

15. 2024. Performance Standards for Antimicrobial Susceptibility Testing, 34th Edition. Clinical and Laboratory Standards Institute, Wayne, PA.
